# Hungry Bone Syndrome in a Female Patient With Parathyroid Carcinoma Masquerading As a Thyroid Nodule

**DOI:** 10.7759/cureus.69974

**Published:** 2024-09-23

**Authors:** Manohar V Reddy, Ranjan Basu, Dinesh K Dhanwal, Balaji Balasubramanian, Adhithya N Balaji

**Affiliations:** 1 Internal Medicine, NMC Specialty Hospital, Abu Dhabi, ARE; 2 Diabetes and Endocrinology, NMC Specialty Hospital, Abu Dhabi, ARE; 3 Surgery and Oncology, NMC Specialty Hospital, Abu Dhabi, ARE; 4 Oncology, Università Cattolica Del Sacro Cuore, Rome, ITA

**Keywords:** hungry bone syndrome, hypocalcemia, osteoporosis, parathyroid carcinoma, thyroid nodules

## Abstract

Hungry bone syndrome (HBS) is a rare but severe complication following parathyroidectomy, characterized by profound hypocalcemia and increased bone mineralization. We present a unique case of HBS in a female patient initially diagnosed with a thyroid nodule, which was later diagnosed as parathyroid carcinoma. This case underscores the importance of considering HBS in patients undergoing parathyroidectomy, especially when preoperative diagnosis involves other endocrine pathologies such as thyroid nodules.

## Introduction

Hungry bone syndrome (HBS) is a widely acknowledged complication following parathyroidectomy, characterized by severe hypocalcemia and hypophosphatemia. It occurs due to the rapid uptake of calcium and phosphorus by metabolically active bones deprived of excessive parathyroid hormone (PTH). Clinical features include hypocalcemia, muscle cramps, fatigue, weakness, tetany, and paresthesia. Bone pain may occur due to rapid bone remineralization. Diagnosis involves identifying low serum calcium, phosphate, and magnesium levels, along with elevated bone markers (alkaline phosphatase) after surgery. Electrocardiogram changes (prolonged QT interval) may also be present. Treatment focuses on aggressive calcium and vitamin D supplementation, along with phosphate and magnesium replacement, to restore electrolyte balance. Regular monitoring of serum electrolytes is crucial to prevent complications like seizures or cardiac arrhythmias. We report a case of HBS in a female patient with a preoperative diagnosis of a thyroid nodule, ultimately attributed to parathyroid carcinoma. 

It was previously analyzed that around 13% of cases of HBS occurred after parathyroidectomy for primary hyperparathyroidism, but recent case series report rates as low as 4%. One specific case from a patient population in Saudi Arabia likely reflects a rate closer to the recent studies in the U.S. [1]. However, some studies show a much higher rate, up to 87%, in a cohort of patients from an Asian population [2]. This significant variation may be attributed to better access to healthcare and earlier diagnosis, which allows for timely treatment of hyperparathyroidism, potentially reducing the prevalence of HBS.

For cases following parathyroidectomy due to secondary hyperparathyroidism, prevalence rates range from 20% to 70% [2-4]. Data on tertiary hyperparathyroidism is limited. In one prospective study, the prevalence of HBS in an Indian cohort of thyrotoxic patients post-thyroidectomy was approximately 39%. Hypocalcemia unrelated to post-surgical hypoparathyroidism was also observed in a Singaporean study of postoperative thyroidectomy patients, with a rate of 53%. However, the study did not clearly determine whether these cases were HBS [5-7]. Regarding HBS in metastatic prostate cancer, only case reports are available [6].

## Case presentation

A 19-year-old Filipino lady presented with a sensation of a lump in her neck. The patient reports feeling discomfort or mild pain in the neck area but not constantly, just occasionally. This could be due to pressure from the mass, inflammation, or other underlying causes. Thyroid function tests were normal, but imaging studies identified a moderately suspicious thyroid nodule: a solid, 3.4 cm hypoechoic mass in the right thyroid lobe, classified as TR 4. Fine-needle aspiration (FNA) biopsy is positive for malignancy (category VI, papillary carcinoma).

Microscopy

Hypercellular smears were arranged in many small papillaroid and large true papillary structures with thin vascular stalks and overlapping nuclei, scattered single cells, microfollicles, and flat groups. The follicular cells enlarged round to ovoid nuclei with nuclear salt pepper chromatin, hyperchromasia, occasional visible nucleoli molding, and few intranuclear pseudo-inclusions with granular to dense cytoplasm without clear colloid in the background. Surgical intervention was planned for total thyroidectomy.

During the surgery, the surgeon identified an abnormally large nodule in the right lobe measuring 3.5 cm, with minimal extrathyroidal extension to strap muscles and no gross nodes. PTH monitoring demonstrated a low value of 0.95 pmol/L (1-7 pmol/L), but surprisingly her calcium levels were high.

Postoperative course

The patient developed symptoms of hypocalcemia postoperatively, leading to tetany and altered mental status. Blood tests showed her calcium was surprisingly high (12.17 mg/dL). PTH level was 0.99 pmol/L (low). Her symptoms were present despite calcium levels being in the normal range. Further investigation was carried out. We requested the lab to run a PTH test in the pre-operative sample. It showed a very high PTH (290 pmol/L). It confirmed the presence of hidden hyperparathyroidism with hypercalcemia. Further hyperparathyroidism workup was done. Alkaline phosphate was high. Bone densitometry showed significant osteoporosis (Table 1).

**Table 1:** Postoperative blood tests PTH, parathyroid hormone

Test	Result
Total calcium	12.17 mg/dL (9-10.5 mg/dL)
PTH	0.99 pmol/L (1-7 pmol/L)
Phosphorus	0.99 mg/dL (2.3-4.7 mg/dL)
Magnesium	1.66 mg/dL (1.65-2.6 mg/dL)
Albumin	3.79 mg/dL (3.4-5.4mg/dL)
Alkaline phosphatase - bone specific	1110 ug/L (8.8-21 ug/L)
Serum alkaline phosphatase	2711 (<130 U/L)

Bone densitometry

L spine: age Z-score adjusted for HAZ: -3.70; left hip: age Z-score adjusted for HAZ: -5.12; left forearm: age Z-score adjusted for HAZ: -9.26. Her calcium dropped rapidly in the next two days. Dropped from 12.17 mg/dL to 9 mg/dL (normal: 9-10.5 mg/dL). Phosphorus (1 mg/dL; 2.3-4.7 mg/dL) and magnesium (1.54 mg/dL; 1.65-2.6 mg/dL) were low. So possibility of HBS secondary to hyperparathyroidism and parathyroid carcinoma was considered.

Initial biopsy

The report was suggestive of papillary thyroid carcinoma. The right large nodular structure, surrounded by variably thickened fibrous capsules, consists of diffuse proliferation of epithelial cells with granular cytoplasm and pleomorphic nuclei featuring focal prominent nucleoli. These cells are arranged in solid and trabecular patterns, with occasional small foci of necrosis (post-FNA) and infiltrating fibrous capsules. Additionally, small nodules are formed, separated by somewhat fibrous bands, with suspicion of invagination of tumor cells into the blood vessels of the capsule. No infiltration into surrounding thyroid parenchyma, mitotic figures, or perineural or lymphatic invasion is identified.

In view of new clinical findings, we sent histopathology slides for a second opinion and further immunohistochemical tests to the Mayo Clinic. Reports confirmed parathyroid carcinoma, oxyphilic type. Complete nuclear parafibromin loss is detected, which correlates with HRPT2 (CDC73) mutation and also parafibromin deficient parathyroid neoplasm. 

Treatment

It included intravenous calcium gluconate, intravenous magnesium, and oral calcitriol. Her symptoms gradually improved with intravenous infusion. We continued infusion for the next 72 hours and then tried to taper the calcium infusion and continue with oral calcium supplements. However, her calcium dropped within 24 hours after stopping intravenous calcium. We restarted the calcium infusion and made an attempt to transition to oral calcium. However, serum calcium levels could not be maintained. We added indapamide 1.5 mg and increased the dose of oral calcium to its maximum oral dosage. Increased calcium-rich diet. However, none of them could maintain the calcium levels. We had to continue intravenous calcium infusion for two weeks. Then our plan was to start Natpara (PTH) but it was not available. So the next best option was to start on Teriparatide 20 mcg. She was given subcutaneous injections twice daily. Within the next two days, her calcium stabilized. We could taper and stop the calcium gluconate infusion. Then we discharged her with oral medicines (Cholecalciferol 400 IU and calcium as calcium carbonate 600 MG two tablets three times a day) and an injection of Teriparatide 20 mcg once daily. Follow-up as outpatient showed calcium levels were maintained. Gradually we reduced the dose of Teriparatide and stopped over a period of one month.

## Discussion

HBS is a rare but potentially severe complication of parathyroidectomy, especially in cases of parathyroid carcinoma. Hypocalcemia after parathyroidectomy is generally transient when the bone disease is mild. However, in some patients with end-stage kidney disease, postoperative hypocalcemia can be severe and prolonged, despite normal or elevated levels of PTH. This complicates with postoperative care [6-10]. The atypical presentation of a thyroid nodule in our case underscores the importance of considering alternative endocrine pathologies and thorough exploration during surgery. A pre-operative nomogram can be very helpful in predicting the possibility of HBS (Table 2) [11-13]. 

**Table 2:** Predictors of HBS HBS, hungry bone syndrome

Pre-operative nomogram to predict for HBS [13]
Higher pre-operative PTH levels
Higher bone alkaline phosphatase
Heavier total weight of resected parathyroid gland
Radiologic evidence of bone disease
Lower pre-operative corrected calcium in case of secondary hyperparathyroidism due to renal origin

The pathophysiology of HBS involves excessive calcium and phosphate deposition in bones, leading to prolonged hypocalcemia. Management includes aggressive calcium and vitamin D supplementation, along with close monitoring of electrolytes.

## Conclusions

This case highlights the complexity of managing a 19-year-old female patient with concurrent papillary thyroid carcinoma and parathyroid carcinoma (oxyphilic type). The patient initially presented with a suspicious thyroid nodule and underwent total thyroidectomy based on a diagnosis of papillary carcinoma. Postoperatively, she developed unexpected hypercalcemia, which, combined with further laboratory testing and imaging, revealed underlying hidden hyperparathyroidism and significant osteoporosis.

The histopathological confirmation of parathyroid carcinoma necessitated an intensive postoperative management plan to address her severe hypocalcemia, which was complicated by HBS. Despite multiple attempts to stabilize her calcium levels with intravenous calcium, oral supplements, and medications, her condition required the use of Teriparatide, which ultimately helped maintain calcium levels and allowed for successful weaning off intravenous calcium.

This case underscores the importance of considering coexisting parathyroid pathology in patients with complex calcium metabolism disorders and the need for a multidisciplinary approach to manage postoperative complications effectively.
